# Membrane Binding and Self-Association of the Epsin N-Terminal Homology Domain

**DOI:** 10.1016/j.jmb.2012.08.010

**Published:** 2012-11-09

**Authors:** Chun-Liang Lai, Christine C. Jao, Edward Lyman, Jennifer L. Gallop, Brian J. Peter, Harvey T. McMahon, Ralf Langen, Gregory A. Voth

**Affiliations:** 1 Department of Chemistry, Institute of Biophysical Dynamics, James Franck Institute, and Computation Institute, University of Chicago, 5735S Ellis Avenue, Chicago, IL 60637, USA; 2 Zilkha Neurogenetic Institute, University of Southern California, 1501 San Pablo Street, Los Angeles, CA 90033, USA; 3 Department of Physics and Astrophysics, and Department of Chemistry and Biochemistry, University of Delaware, Newark, DE 19716, USA; 4 MRC Laboratory of Molecular Biology, University of Cambridge, Hills Road, Cambridge CB2 2QH, UK

**Keywords:** ENTH, epsin N-terminal homology, PIP_2_, phosphatidylinositol 4,5-bisphosphate, CG, coarse-grained, MD, molecular dynamics, CME, clathrin-mediated endocytosis, N-BAR, N-terminal Bin/Amphiphysin/Rvs-homology, EM, electron microscopy, HAS, hybrid analytical systematic, PMF, potential of mean force, RT, room temperature, epsin, ENTH domain, molecular dynamics, membrane remodeling, amphipathic helix binding

## Abstract

Epsin possesses a conserved epsin N-terminal homology (ENTH) domain that acts as a phosphatidylinositol 4,5-bisphosphate‐lipid‐targeting and membrane‐curvature‐generating element. Upon binding phosphatidylinositol 4,5‐bisphosphate, the N-terminal helix (H_0_) of the ENTH domain becomes structured and aids in the aggregation of ENTH domains, which results in extensive membrane remodeling. In this article, atomistic and coarse-grained (CG) molecular dynamics (MD) simulations are used to investigate the structure and the stability of ENTH domain aggregates on lipid bilayers. EPR experiments are also reported for systems composed of different ENTH-bound membrane morphologies, including membrane vesicles as well as preformed membrane tubules. The EPR data are used to help develop a molecular model of ENTH domain aggregates on preformed lipid tubules that are then studied by CG MD simulation. The combined computational and experimental approach suggests that ENTH domains exist predominantly as monomers on vesiculated structures, while ENTH domains self-associate into dimeric structures and even higher‐order oligomers on the membrane tubes. The results emphasize that the arrangement of ENTH domain aggregates depends strongly on whether the local membrane curvature is isotropic or anisotropic. The molecular mechanism of ENTH‐domain-induced membrane vesiculation and tubulation and the implications of the epsin's role in clathrin-mediated endocytosis resulting from the interplay between ENTH domain membrane binding and ENTH domain self-association are also discussed.

## Graphical Abstract

**Figure:**
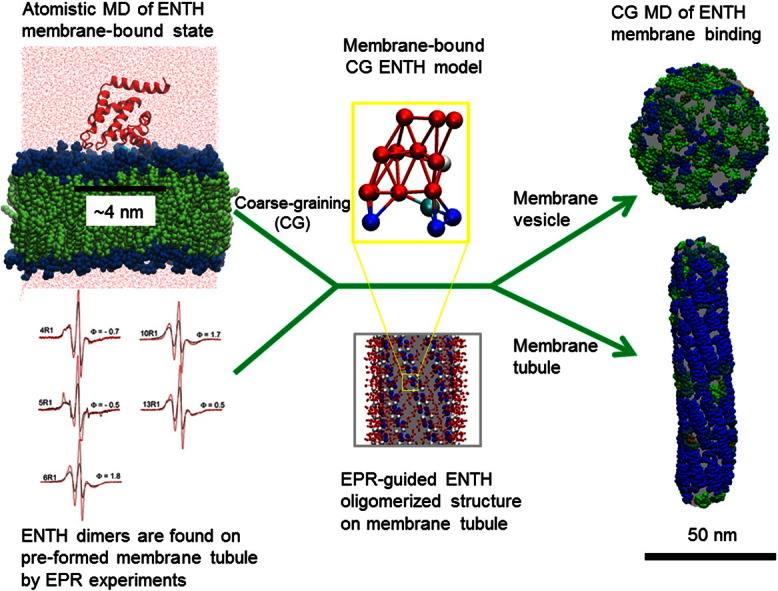

**Highlights:**

► ENTH domain plays an essential role in clathrin-mediated endocytosis. ► Membrane binding of ENTH domain is studied by a combined MD and EPR approach. ► A CG ENTH model for CG MD simulations is developed. ► ENTH-bound membrane tubules and vesicles are studied via CG MD simulations. ► The arrangement of ENTH domains highly correlates with local membrane curvature.

## Introduction

The cytoplasmic membrane surface serves as a platform for many critical cellular signaling and trafficking pathways. Specific membrane targeting by proteins and dramatic changes in the shape and topology of the membrane are often involved in these complex cellular processes, which require coordinated efforts between proteins and lipids in a spatially and temporally precise manner.^1–5^ Clathrin-mediated endocytosis (CME) is one of the essential cellular processes that requires the coordinated action of multiple membrane proteins functioning together.^6,7^ The invagination of the clathrin-coated pit is an energetically costly step in this process involving several proteins, including epsin,^8–10^ that come into direct contact with and bind to the membrane surface. Accumulating evidence suggests a dual role for epsin of inducing membrane curvature and recruiting accessory proteins in the early stage of CME^11,12^; it contains multiple conserved binding motifs that can interact with several accessory proteins associated with CME, for example, AP2, Eps15, clathrin, and ubiquitinated proteins.^13,14^ In addition, it has been recently shown that epsin is required for clathrin-coated vesicle scission.^15^

An important feature of epsin is its highly conserved amino-terminal domain, known as the epsin N-terminal homology (ENTH) domain. The ENTH domain directs epsin to the membrane surface and can induce membrane remodeling.^16^ The membrane targeting and remodeling ability of the ENTH domain relies on a specific interaction with phosphatidylinositol 4,5-bisphosphate (PIP_2_) and the insertion of an N-terminal amphipathic helix (denoted helix zero, H_0_) into the inner leaflet of the plasma membrane.^16–19^ In Ford *et al.*,^16^ the ENTH domain structure—including H_0_—was resolved by co-crystallizing with d-myo-inositol-1,4,5-triphosphate (IP_3_) where it was observed that the three phosphate groups of IP_3_ have interactions with up to eight correspondingly positively charged residues in the ENTH domain. Directed by the crystal structure, mutagenesis demonstrated the importance of the L6 residue of H_0_ in membrane remodeling. In the absence of the PIP_2_ headgroup analogue, however, the H_0_ region was disordered and was not resolved in the crystal structure. These data highlight that targeting to PIP_2_ and structuring of H_0_ are critical to the ENTH domain's function in membrane binding and membrane remodeling.

Amphipathic helices—α-helices with a hydrophobic and a hydrophilic face—are a quite general mechanism by which proteins target membrane surfaces. The membrane surface and, in particular, defects in the membrane surface drive folding of the helix and partitioning at the hydrophobic/hydrophilic interface within the membrane.^20–24^ Upon binding to membrane, the shallow penetration of amphipathic helix leads to the generation of local spontaneous curvature through the displacement of the lipid headgroups and reordering the surrounding lipid tailgroups (the “wedge” mechanism), with the depth of insertion shown to be a crucial determinant^25,26^ of curvature induction.^1,4,27,28^ Importantly, the shallow hydrophobic insertion of ENTH H_0_ has been shown to be sufficient to drive membrane fission and hence transform continuous membranes into divided ones, such as the final vesicle scission process in CME.^15^

It is instructive to compare the structure of the ENTH domain with another class of membrane remodeling modules, namely, N-terminal Bin/Amphiphysin/Rvs-homology (N-BAR) domains, which are present in both amphiphysin and endophilin proteins.^29–32^ The N-BAR dimers are crescent shaped with several positively charged residues on the concave surface. The N-terminal amphipathic helix embeds in between the head and tail regions of lipids of the contacting monolayer when the N-BAR domain binds the membrane. Experiments have demonstrated that the concave shape of the BAR domain, the positively charged residues on the concave surface, and the embedded N-terminal amphipathic helices are all critical factors for membrane remodeling.^29,33–39^ How each of these structural features work together to drive membrane deformation has been a topic of recent interest experimentally,^1,4,5,27,40,41^ with atomic-level molecular dynamics (MD) simulation,^42–44^ coarse-grained^45–48^(CG) MD, and mesoscopic simulation.^49,50^

In contrast to the crescent shape of the N-BAR dimer, the ENTH domain has no obvious crescent‐shaped structure and instead is more compact and “block-like”; how this compact structure can impose curvature by the scaffold mechanism is not immediately apparent. Notably, incubation of the ENTH domain with liposomes (~ 300–400 nm in diameter) containing PIP_2_ induces both tubulation and vesiculation.^16^ Based on previous insights from N-BAR domain membrane remodeling, it is plausible that the membrane remodeling ability of the ENTH domain may be correlated to both the insertion of the H_0_ helices in the membrane,^4^ as well as the collective behavior of ensembles^30,49^ of ENTH domains. However, the detailed molecular mechanism and the factors that control, for example, the formation of larger ENTH domain aggregates, the structure of those aggregates, and how ENTH domain aggregates induce membrane remodeling and deform membranes into different morphologies remain unclear.

Different remodeling pathways lead to different membrane morphologies (e.g., membrane vesicles and tubes). The distinguishing feature of vesicles (compared to tubes) is an isotropic curvature, which can arise from randomly oriented amphipathic insertions.^50^ In contrast to vesiculation, stabilization of membrane tubes demands an anisotropic spontaneous curvature, which can arise by imposition of a tightly packed scaffold (as in the F-BAR^30^) or by aligning amphipathic modules (such as the H_0_ helix) that induce a locally anisotropic spontaneous curvature. But what molecular details determine the pathway followed by ENTH domains? In particular, what is the role of PIP_2_ binding, and how does this control the formation of ordered *versus* disordered ENTH aggregates? The experiments by Yoon *et al.* suggested that aggregation and ordered arrangement of ENTH domains play roles in ENTH‐domain-induced membrane remodeling.^51^ However, due to the lack of molecular-level resolution of the system, it remains unclear how membrane binding and self-association of ENTH domains lead to membrane remodeling.

An approach that combines the strengths of site-directed spin labeling and EPR spectroscopy with atomistic/CG MD simulations is taken in the present work in order to study in detail the aforementioned mechanisms and unresolved issues. EPR spectroscopy has become a powerful tool in elucidating structural features of proteins^52–54^; for example, it has provided important molecular-level details for the membrane‐penetrating depth and membrane-induced ordering of the N-terminal helix insertions of the ENTH domain^19^ and other membrane remodeling proteins.^34^ Moreover, EPR spectroscopy can be used to determine inter-residue distances.^53,55–57^ In favorable cases, this information provides sufficient constraints to generate three-dimensional structures of proteins with atomic resolution.^54^ Distance information can also provide detailed insights regarding the spatial arrangement of oligomerized proteins.^58^

Similar to EPR spectroscopy, atomistic MD simulations can provide quantitative information at atomic-level resolution (e.g., averaged penetration depths of inserted helices and atomic-level details of membrane–protein binding interface). Thus, the simulations can be directly related to quantities that are measurable by EPR spectroscopy, validating the simulation data and providing confidence in the features observed in the simulations. Beyond atomic‐level information, insight into membrane binding and self-association of ENTH domains at long length and time scales is also invaluable. However, this poses a significant challenge since it remains computationally too expensive to simulate with atomistic MD more than a few membrane-bound ENTH domains. CG simulations, on the other hand, allow access to longer length and time scales by reducing the number of degrees of freedom that must be simulated, but they require careful parameterization to obtain a CG model that can faithfully capture the quantitatively (or even qualitatively) correct physics of the system. Several CG methods and protocols have been developed and are specifically suitable for the systems related to protein-induced membrane remodeling.^59,60^ Here, we take a systematic approach to ENTH domain CG model development.

In this article, we present EPR data showing that the N-terminal helix of ENTH (H_0_) becomes structured when ENTH domain binds to PIP_2_-containing membrane. The EPR penetration depth measurements show that H_0_ embeds at the level of lipid phosphate groups, the optimal location for H_0_ to function as a “wedge”, leading to membrane curvature generation.^27^ Furthermore, EPR data acquired in different environments (vesicles and preformed membrane tubes) suggest different oligomeric states of the bound ENTH domains. Atomistic and CG MD simulations in turn provide molecular-level insight into membrane binding and self-association of ENTH domains. Atomistic MD data are supported by the EPR experiments and show that the ENTH domain H_0_ is stable as an α‐helical structure and locates at the level of the lipid phosphate groups. Importantly, the MD data provide additional atomic‐level details of the membrane–protein binding interface.

The self-association of hundreds of ENTH domains on different membrane surfaces was then studied by CG MD simulations. The membrane docking geometry of the ENTH domain and membrane–ENTH domain interactions observed in atomistic MD simulations was incorporated in the CG ENTH model. The ENTH CG model and a previously developed CG lipid model^61^ are used to investigate how ENTH domains bind and self-associate onto two membrane morphologies commonly observed from ENTH‐domain-induced membrane remodeling: vesiculated and tubulated structures. The higher‐order oligomeric structures inferred from the EPR data were incorporated into the CG model to investigate the stability of the putative ENTH oligomers in the different environments and their effect on tubule morphology. Overall, the hybrid approach supports the idea that ordered ENTH domain packing (dimeric structure) favors a tubular membrane structure, and randomly oriented ENTH domain monomers dominate vesiculated structures. Furthermore, molecular‐level information from CG MD simulations suggests that preformed membrane tubules stabilize ordered ENTH lattices, helping to rationalize the EPR data, and it leads us to suggest possible molecular mechanisms of ENTH‐domain-induced membrane vesiculation and tubulation.

## Results

### Atomistic MD simulation study of ENTH domain membrane-bound state

**Fig. 1:**
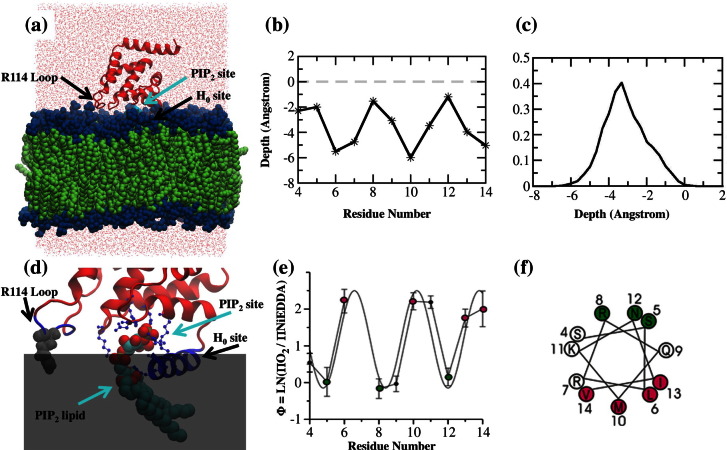
(a) Atomistic MD simulation of the ENTH domain in a lipid bilayer. Representative snapshots are taken from MD simulation of the complex composed of an ENTH domain and a bilayer containing one PIP_2_ molecule. The ENTH domain is represented as a red ribbon. Lipid headgroups are shown in blue and lipid tails are shown in green. Two black arrows indicate the locations of the R114 loop and H_0_ helix site. The PIP_2_-bound site of the ENTH domain is indicated by a cyan arrow. Red points represent oxygen atoms of waters in the MD system. (b) The average insertion depth of C^α^ from residues 4 to 14 of helix H_0_ is shown. Depth is measured parallel to the membrane normal with respect to the lipid bilayer phosphate groups. (c) Distribution of the center of mass of H_0_ with respect to the lipid bilayer phosphate groups as was calculated from the last 50‐ns trajectory of MD simulation. (d) Close-up view of the ENTH domain membrane-interacting motifs and the binding site of the PIP_2_ headgroup to the ENTH domain. A gray transparent shadow is used to illustrate the location of lipid bilayer. The protein backbone is shown as a red ribbon, except for the R114 loop and H_0_ site, which are highlighted in blue. Residue R114 and a PIP_2_ molecule are explicitly included, shown in sphere representation. The eight residues indicated in Ford *et al.*^16^ that directly interact with the PIP_2_ headgroup are shown in a blue ball-and-stick representation. (e) Accessibility measurements indicate the formation of an amphipathic helical structure in the N-terminus of the epsin 1 ENTH domain. (e) shows the contrast parameter Φ as a function of residue number. Note that higher values of Φ correspond to deeper C^α^ insertion in (b). The period of oscillation is indicative of helical structure as indicated by the sinusoidal line drawn with an ideal periodicity of 3.6 amino acids. (f) Local maxima (magenta circles) fall onto the hydrophobic face and local minima cluster on the hydrophilic side of the helical wheel representation shown in (f).

To study the details of the membrane‐bound state, and to provide data for the parameterization of a CG model of membrane binding of the ENTH domain, we performed atomistic MD simulations of the ENTH domain membrane-bound state (Fig. 1a). In Fig. 1b, H_0_ residue-to-residue membrane penetration depth measured from atomistic MD shows good agreement with EPR measurement (see Fig. 1e and f and also Supplementary Data) by measuring the relative penetration depth of each H_0_ residue with respect to the lipid bilayer phosphate groups. Also, the center of mass distribution of H_0_ from atomistic MD data indicated that H_0_ embeds at the level of lipid bilayer phosphate groups, as shown in Fig. 1c. It should be mentioned that in EPR experiments (Fig. 1e) based on the calibration with Φ values as described in Materials and Methods, the average immersion depth of the lipid-facing R1 side chains (Φ maxima) is on the order of 8 Å (see also Supplementary Data). Taking into account that the nitroxide moiety of the R1 side chain is typically within 7–10 Å of the center of the helix,^62^ the center of the H_0_ helix is also at the level of the lipid bilayer phosphate groups. Overall, the atomistic MD simulation agrees quantitatively with the EPR data on the amphipathic behavior of the H_0_ helix in membrane binding. Also, the averaged location of the H_0_ helix at the level of the lipid bilayer phosphate groups implies that the H_0_ helix is likely to exert a membrane “wedge” effect, leading to membrane curvature generation.

Importantly, the atomistic MD data offer additional molecular details at the ENTH domain-bilayer binding interface, as well as provide data used to construct the CG ENTH model. Atomistic MD simulation identifies three regions with important ENTH domain–membrane interactions: the PIP_2_ binding pocket, the arginine 114 loop (R114 loop), and H_0_ (Fig. 1d). The MD data show that the headgroup of the PIP_2_ lipid is quite stable in the binding pocket, as indicated by the specific interactions between positively charged residues and the 4- and 5-phosphate groups of the PIP_2_ headgroup. Such stable binding might help the ENTH domain to effectively respond to the PIP_2_ signal from the membrane and prolong the binding of ENTH domain on the membrane surface. Recent single‐molecule studies have examined the diffusive behavior of ENTH domains on membranes containing PIP_2_, and the data show that ENTH domains exhibit about half of the diffusion rate compared to the diffusion rate of the bulk lipids.^63^ This observation implies that ENTH might diffuse in membrane as an ENTH–PIP_2_ complex. Additional drag from the inserted H_0_ might explain why the ENTH domain diffuses slower than the bulk lipids.

The specific coordination between positively charged residues in the binding pocket to the PIP_2_ lipid headgroup may also explain why H_0_ stabilizes at the lipid bilayer interface region. Three positively charged residues (R7, R8, and K11) on H_0_ are involved in coordination of phosphate groups of PIP_2_. R7 and R8 interact with 1- and 4-phosphate groups of PIP_2_, respectively. This observation suggests that the position of H_0_ is tightly coupled to the position of the PIP_2_ headgroup, though it remains unclear exactly how H_0_ couples PIP_2_-specific binding to membrane insertion.

The interaction between the R114 loop and the membrane surface is also observed and this provides an additional contact that orients and stabilizes the bound protein. R114 is located in an unstructured loop region between helix 6 (residues 100 to 108) and helix 7 (residues 121 to 134). It is seen in the MD simulations that R114 frequently interacts with lipid phosphate groups and PS lipid headgroups, suggesting a nonspecific electrostatic interaction. This observation implies that R114 contributes to targeting and binding. Moreover, the R114 loop also acts as a support in addition to H_0_ and helps to stabilize the ENTH domain membrane docking geometry. Indeed, previous experiments have reported that the R114A mutant exhibits a 4-fold increase of dissociation rate constant compared to the wild‐type ENTH domain; however, this mutant has no major effect on monolayer penetration.^18^ Taken together, our atomistic MD data are consistent with the experimental data, thus validating the use of a simplified membrane model in the CG context and offering insight into key interactions that need to be considered when constructing the CG model.

### Structural features of H_0_ when ENTH domains are bound to preformed tubules

**Fig. 2:**
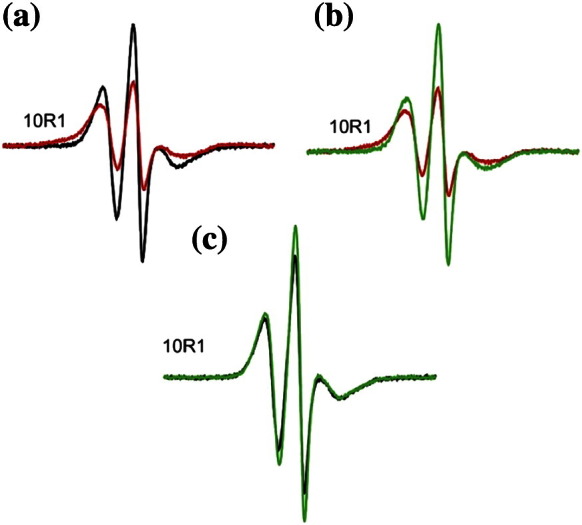
EPR evidence for dimerization of the epsin 1 ENTH domain. The EPR spectra of 10R1 incubated with preformed tubules (red) or liposomes (black) are overlaid in (a). Preformed tubule‐bound 10R1 exhibits pronounced line broadening indicative of spin–spin interaction. To investigate the effect of spin interaction on the line shape, we incubated 10R1 with preformed tubules in the presence of twofold excess of unlabeled protein, resulting in a spectrum with reduced line broadening and increased amplitude [green trace in (b)]. (c) shows the analogous dilution experiment as shown in (b) with the exception that liposomes were used. The scan width for all spectra is 150 Gauss and all spectra are normalized to the same number of spins.

EPR data for spin‐labeled derivatives of ENTH in solution and bound to liposomes establish that H_0_ becomes ordered and forms an amphipathic α-helical structure upon incubation with liposomes (see Fig. 1e and f and Supplementary Data). We examined whether similar conformational changes might also occur when ENTH domains are bound to preformed tubules. Toward this end, preformed tubules were prepared according to previously published methods^64^ and the formation of predominantly tubular structures was confirmed by negative‐stain electron microscopy (EM) using uranyl acetate. As illustrated with the example of 10R1 (Fig. 2a, red trace), binding to preformed tubules causes pronounced spectral changes, indicating that the N-terminal region also becomes ordered under these conditions. However, there are some clear differences, when compared to the EPR spectra obtained in the presence of liposomes (Fig. 2a, black trace). The most pronounced difference is the overall line broadening and the concomitant loss in spectral amplitude. Such changes are often indicative of strong dipolar interaction between spin labels (< 20 Å distance). In the present case, any spin–spin interaction would have to be intermolecular in nature, as only one site (position 10) was labeled in each protein. To investigate this possibility, we tested whether a dilution with unlabeled ENTH domain (33% labeled protein and 67% unlabeled protein) could alleviate the spectral broadening. Indeed, such spin dilution causes significantly sharper lines (Fig. 2b, green trace), yielding a spectrum remarkably similar to that of 10R1 incubated with liposomes. It is well established that the comparison of spectra with and without spin–spin interaction can be used to evaluate an effective broadening function and determine an interlabel distance distribution.^52–58,65^ Typically, distances are obtained under frozen conditions, but it has been demonstrated that distances can also be estimated with reasonable accuracy provided that the interspin vector does not tumble rapidly.^56^ This condition is met for membrane-bound proteins and, using such an analysis (see Materials and Methods), we obtain a distance distribution centered on 13 Å. Thus, under these conditions, the ENTH domain predominantly forms dimers (or potentially even higher‐order oligomers).

Next, we performed analogous spin-dilution experiments with 10R1 that had been incubated with liposomes (Fig. 2c). Also, in this case, an effect of spin dilution could be observed; however, the extent of the dilution-induced spectral change is much smaller. Spectral analysis suggests that, under these conditions, only a subset of the 10R1 derivatives (~ 10% to 20%) undergoes spin–spin interaction, suggesting that the aforementioned dimerization is strongly reduced.

**Fig. 3:**
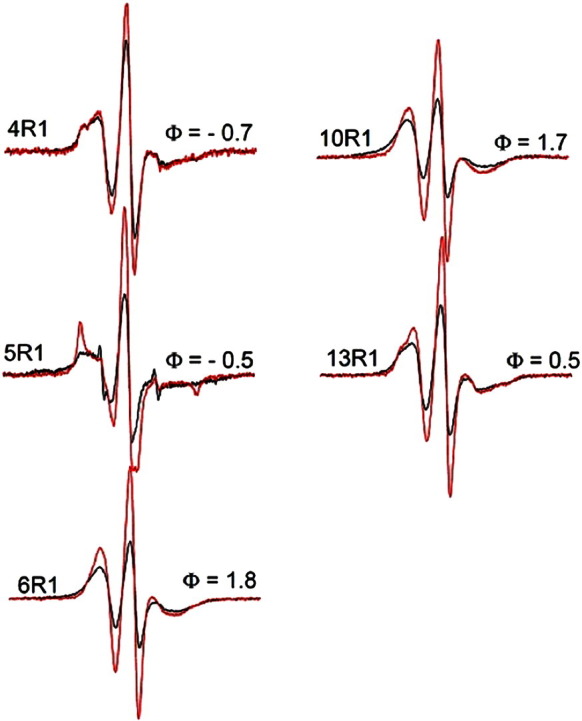
EPR spectra for selected epsin 1 ENTH domain spin‐labeled derivatives bound to preformed tubules. The black spectra are obtained from proteins labeled at the indicated positions while the red spectra were obtained using the indicated spin‐labeled derivatives diluted with twofold excess of unlabeled protein.

To further investigate the structural features of ENTH domains when bound to preformed tubules, we recorded the EPR spectra of additional selected sites in the N-terminal region. As shown in Fig. 3, all of the sites tested revealed the presence of spin–spin interaction. From analysis of the spectra in Fig. 3, we find distances of 13 Å and 15 Å for the 6R1 and 13R1 derivatives, respectively. The distance for 4R1 is larger, with a broad distance distribution centered on 21 Å. In the crystal structure, this position faces back toward the protein and the longer distance is likely due to the fact that this residue is facing away from the dimerization interface. In agreement with this notion, we find that the adjacent residue 5, which is projecting into the opposite direction, has the shortest distance of 9 Å. Very similar distances were obtained in the frozen state (data not shown). Interestingly, the EPR spectrum of the spin-diluted form of the 5R1 derivative indicates substantial immobilization. Such pronounced immobilization is indicative of significant packing interactions, further indicating that this residue is likely to be at or near the contact surface between adjacent proteins. Collectively, all of these data support the notion that ENTH domains can dimerize in a manner that brings the N-terminal regions from two proteins into close proximity. To further validate the membrane insertion of H_0_ under these conditions, we next determined the depth parameter Φ for each of these derivatives. Positive Φ values were obtained for all sites located on the hydrophobic face of helix 0 while negative Φ values were observed for positions 4 and 5. These data are consistent with the notion that H_0_ becomes helical with one membrane embedded and one solvent‐exposed face.

### Development of the CG ENTH model

**Fig. 4:**
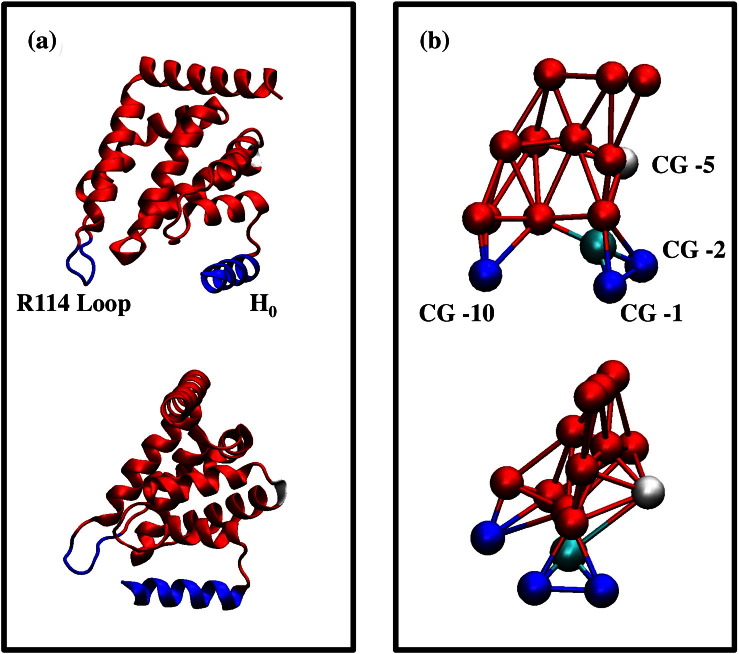
All-atom structure of the ENTH domain (a) and the 16‐site CG ENTH domain model (b). The two N-terminal amphipathic helix sites [CG-1 and CG-2 in (b)] and one R114 loop site [CG-10 in (b)] are shown in blue. The PIP_2_‐interacting site is shown in cyan. The CG site 5 [CG-5 in (b)] that consists of two solvent-exposed hydrophobic residues (V50 and V51) is shown in white. The rest of the domain is in red.

By combining the EPR data on inter-ENTH domain contacts with our atomistic MD simulation data, we developed a CG model to study ENTH domain binding and self-association. The CG ENTH monomer model (Fig. 4) retains the important membrane‐interacting features of the ENTH domain observed in the atomistic MD simulations, while at the same time reducing the degrees of freedom of the system so longer length and times scales can be sampled. The details of the coarse-graining procedures and considerations of cross-interaction of CG systems (e.g., lipid–lipid, lipid–protein, and protein–protein interactions) are described in Materials and Methods.

### CG ENTH multimer model

**Fig. 5:**
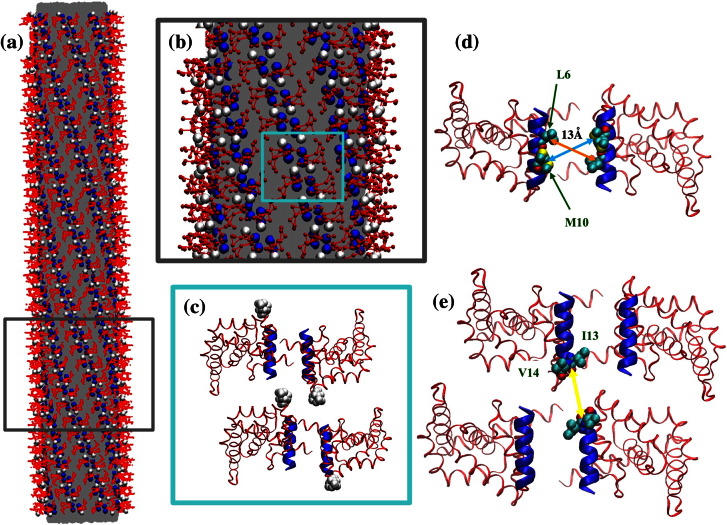
CG MD simulation system with an ENTH-lattice‐coated membrane tubule. (a) A membrane tubule is shown in gray. Red lines connect CG sites. Selected CG sites are shown in sphere representation. H_0_ and hydrophobic contact sites V50 and V51 are shown in blue and white spheres, respectively. (b) Close-up of (a). (c) Close-up ribbon view of the ENTH tetramer in (b). Four ENTH domains are shown in red ribbons, while H_0_ helices are shown in thicker blue ribbons. Hydrophobic contact patches, V50 and V51, are shown as white spheres. (d) Bottom view of (c), with only a dimer unit shown. L6 and M10 are shown in cyan spheres. The orange and light blue arrows represent the measured EPR distance (~ 13 Å) for L6 and M10, respectively. (e) Bottom view of (c). Offset packing between two dimers that brings the I13 and V14 region of H_0_ in close proximity as highlighted with a yellow arrow. I13 and V14 are shown in sphere representation. This is indicated by an EPR distance profile of 15 Å for I13 and 10 Å for V14. These distances are shorter than would be expected for an antiparallel dimer pairing.

EPR-derived H_0_–H_0_ distance restraints were applied to the construction of the CG ENTH domain lattice on lipid tubules; the ENTH domain dimer was used as a minimal unit of the ENTH domain lattice. As shown in Fig. 5, the resulting ENTH domain lattice is compact, and the lipid tube is built inside the protein lattice with a diameter of 16 nm. The ENTH domain dimer structure was derived from the EPR experimental data, with the amphipathic helices arranged in antiparallel orientation, also suggested in previous work.^51^ The ENTH domain dimer structure was constructed based on the symmetric EPR distance profiles for residue 6 and residue 10 (~ 13 Å) as shown in Fig. 5d. The offset packing between two dimers was motivated by additional EPR distance restraints, namely, that relatively shorter EPR distance profiles are found for residue 13 and residue 14. The EPR distances are around 15 Å and 10 Å for residue 13 and 14, respectively (Fig. 5e). Both these distances are considerably shorter than what would be expected if they were due to interactions within the dimer between an antiparallel H_0_ pairing. The shorter symmetric distances between residues 13 and 14 suggest the possibility of an extended protein lattice that assembles on the lipid tube structure. Interestingly, a hydrophobic patch is observed in the region of the proposed dimer–dimer contact interface. This hydrophobic patch consists of four valine residues, two from each ENTH domain (V50 and V51, shown in Fig. 5c as white spheres). Forming this dimer–dimer hydrophobic contact arranges the H_0_ helices parallel to the lipid tube axis, aligned so that they work together to induce an anisotropic spontaneous curvature. We emphasize that the CG dimer model and multimer models are constrained by the known orientation of membrane‐bound ENTH and the EPR distances reported here.

### CG MD simulations of ENTH-bound membrane tubules and vesicles

ENTH-bound membrane tubules and vesicles were simulated with CG MD simulations chosen to resemble EPR experimental conditions. These systems were designed to investigate self-association of ENTH domains upon binding to different membrane morphologies. CG MD results show that simulations not only support the EPR observations but also provide additional insights into the system at the molecular level. These molecular‐level details in turn shed light on the mechanism of membrane remodeling induced by ENTH domains. The setup of the CG MD systems for the ENTH-bound membrane tubules (type A and type B tubes) and membrane vesicle systems is described in Materials and Methods.

#### ENTH-bound membrane tube systems

**Fig. 6:**
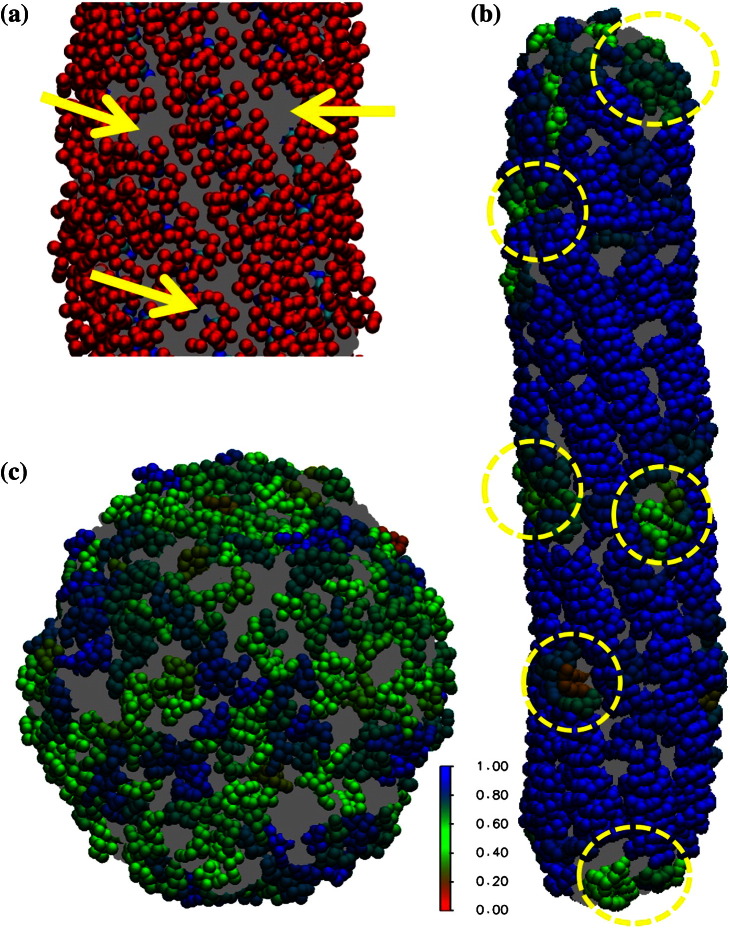
(a) Close-up view of the ENTH domain lattice on a type A tube. Selected defect sites are shown with yellow arrows. Lipids are shown in gray. CG sites are shown as red spheres, except for H_0_ sites that are shown in blue spheres and PIP_2_ interaction sites that are shown in cyan. (b) Local orientational order of ENTH domain-bound membrane tube (type B tube). The simulation snapshot is colored to show the local orientational order in the neighborhood of each ENTH domain. The color scale reflects the average of the unsigned dot product between the H_0_ axes of neighboring ENTH domains within 5 nm. Blue corresponds to a high degree of local order, and red corresponds to local disorder. There is considerably more disorder for both the capping regions, and the defect sites are scattered across the tube in a nonuniform manner. Selected defect sites are highlighted in yellow dashed circles. (c) Local orientational order parameter color map of an ENTH domain-bound membrane vesicle. The representative simulation snapshot is taken from an equilibrated CG MD simulation.

We performed CG MD simulations of ENTH domain-bound membrane tubes in order to investigate our hypothesis for the structure of ENTH oligomers derived from the EPR data on preformed tubules. Our hypothesized contacts lead to a regular, helical coat of ENTH domains, as shown in Fig. 5a. The membrane tube systems are designed to investigate the interplay between the ENTH domain lattice and an anisotropic curvature membrane environment; note that when forming the proposed lattice, the H_0_ helices are optimally aligned, with the long axis parallel to the axis of the tubule. Of interest is how stable the ordered lattice is at non-cryogenic temperatures, as it seems likely that perfect order will not be maintained. While we expect that the tubule environment should favor ordered structures, it is not clear to what extent this order will be preserved. To this end, we calculated a local orientational order parameter to determine the local order of neighborhoods of ENTH domains. The direction of the axis of ENTH H_0_ is calculated, and then the (unsigned) dot product between pairs of ENTH H_0_ helices is averaged over the local neighborhood (defined by a 5‐nm cutoff) of each. In Fig. 6a, it is shown that the overall ENTH domain lattice on type A tube remains stable over the duration of the CG MD simulation and that the averaged local orientational order parameter converges to a value around 0.92, indicating that ENTH domains maintain their local alignment with their neighbors (Fig. S2 shows that the orientational order eventually does not change with time; i.e., the lattice has equilibrated). This suggests that the proposed ENTH domain lattice is compatible with the membrane tube structure.

In the type A (periodically replicated) tube system, CG MD simulations allow a direct observation of how individual ENTH domains self-associate within the ENTH domain lattice at molecular‐level resolution. Information on the self-association at this level of resolution is hard to obtain by either traditional experimental techniques (e.g., EPR and EM) or by computational study via atomic‐level MD simulations given their computational cost. The CG MD data suggest that two factors are important to the stabilization of ENTH domain lattice. First, the formation of the ENTH domain dimer is driven by the dimerization of the H_0_ helices. The ENTH dimer structure potentially provides a large contact interface between adjacent dimers along the direction parallel to the lipid tube axis by further promoting the formation of ENTH domain “dimers of dimers”. The proposed interface between dimers coordinate the H_0_ helices parallel to the lipid tube axis direction so that they exert a strongly anisotropic spontaneous curvature. Second, crowded packing conditions on the membrane tube may also be important to the stabilization of the protein lattice and the kinetics of lattice formation. ENTH domain self-association is a dynamic process—individual ENTH domains and higher‐order aggregates diffuse on the membrane surface and continually adsorb and desorb. The crowded conditions offer an environment where ENTH domains interact with each other frequently, increasing the likelihood of dimer formation prior to desorption. It should be noted that such tightly packed protein lattices with well-defined contacts and interfaces are commonly found in high‐resolution EM of membrane‐tubule‐coated proteins such as endophilin, F-BAR, and dynamin.^30,66,67^ It should be noted, however, that such specific oligomerizaton might not always be required for proteins to tubulate the membrane. For example, α-synuclein tubulates membranes by virtue of a highly anisotropic and long wedge.^25,26^

In order to test the importance of dimer stability to the stability of lattice order, we performed a control simulation in the absence of the interprotein attractive interaction between the H_0_ sites (CG site 1 and CG site 2) (Fig. S2). The result of this simulation clearly shows that the prearranged lattice becomes less ordered. These data emphasize that the stability of the dimer (via pairing of H_0_) is important to the stability of the putative protein lattice and furthermore demonstrate that crowding alone is insufficient to stabilize the protein lattice when the specific interactions between protein motifs (i.e., via H_0_–H_0_ interaction) are lacking. Finally, a previous computational study^68^ has shown that preformed tubules without a protein scaffold such as ENTH and without longitudinal tension are unstable—that is, ENTH is required in the present case to stabilize the tubule morphology.

A type B (not periodically replicated) tube system was simulated to examine how the stability of the ENTH domain lattice affects the stability of a self-contained membrane tube. The results in Fig. 6b show that a prearranged ENTH domain lattice is also stable in such a system. Moreover, the local orientational order parameter analysis helps to identify lattice defect sites (Fig. 6b, yellow dashed circles), regions where the ENTH domains are locally disordered. Defects were found on both type A and type B tubes (see also Fig. 6a). Heterogeneity of this kind complicates ensemble averaged experimental data but is consistent with the notion that “perfect order” is unlikely to be obtained under experimental conditions. Furthermore, within our simulation time scale, although most of the dimers remain stable, the membrane tube becomes slightly distorted.

The observation of defects via CG simulations has led us to examine the heterogeneous nature of the ENTH domain lattice order on type B tubes. Indeed, we found heterogeneity of ENTH domain lattice order between different segments of the membrane tube. The overall local orientational order of type B tube fluctuates at around a stable value of 0.88 after 4M time steps of simulation, indicating convergence of the order parameter. However, as shown in Fig. 6b, it is apparent that the capping regions of the membrane tube are less ordered than the middle region of the membrane tube. Also, the defects scatter across the tube and the distribution of defects is not uniform. This motivated us to examine the local order of ENTH domain lattice for different segments of the membrane tube. We divided the tube into several segments and computed averaged local orientational order parameter for each individual segment. Interestingly, as shown in Table S1, the averaged local orientational order parameter of different segments on the tube are heterogeneous, ranging from 0.84 to 0.92 and from 0.86 to 0.94 between two different choices of segmentation. These data suggest that the order of the ENTH domain lattice is heterogeneous, despite the fact that the simulations start from a perfectly uniform ENTH domain lattice.

### Implications of the lattice heterogeneity on ENTH-bound tubules

The observation of heterogeneity of ENTH domain lattice bears on the experimental results for two reasons. First, the heterogeneous nature of ENTH domain lattice on the tube may reduce the stability of ENTH‐domain-coated membrane tube structure, since the increase of lattice heterogeneity leads to a reduction in the anisotropic spontaneous curvature resulting from the ordered packing of ENTH domains, which frustrates the tubule curvature. This idea is consistent with our experimental observations that most ENTH‐domain-coated tubes are short lived (data not shown), necessitating an experimental design employing preformed tubes in order to study ENTH domain packing. Second, the heterogeneity of ENTH domain lattice also offers an explanation as to why it is very hard to experimentally determine one or few definitive ordered lattice structures beyond the dimeric structure. From our EPR experimental observation (data not shown), we generally found EPR interdimer distance distributions to be broad and subject to batch-to-batch variations, which is in contrast to the more specific intradimer pairing of H_0_.

### ENTH-bound membrane vesicle systems

As described in the Introduction, vesiculation is another pathway that is also commonly found in protein-induced membrane remodeling processes. Under the experimental conditions used in this article, we frequently found that ENTH domains can remodel liposomes (~ 300–400 nm in diameter) into smaller vesiculated structure (less than 50 nm in diameter). The CG MD simulations were thus used to investigate how multiple ENTH domains accommodate this type of membrane morphology. It was found that ENTH domains locally self-organize into less structured aggregates as compared to ENTH-bound membrane tube systems. The extent to which aggregates are ordered can again be quantified by calculating the local orientational order of neighborhoods of ENTH domains. It is not surprising to find that ENTH domains exhibit a smaller averaged value of the local orientational order parameter on vesiculated structures (Fig. 6c and Fig. S3) than on membrane tubules (Fig. 6b and Fig. S2), since randomly oriented amphipathic insertions accommodate the isotropically curved membrane vesicle. It should be noted that a control simulation was also performed that started off with structured ENTH domain dimers randomly arranged on the vesicle surface and was relaxed to a similar configuration in terms of the local orientation order parameter with respect to the original system (Fig. S3). Association and dissociation of ENTH domains into and from aggregates are found in both the original and control simulations, respectively. Overall, the monomer form of ENTH domains, that is, no apparent locally ordered packing, dominates the CG MD membrane vesicle system, an observation that is consistent with observations from EPR experiments that ENTH domain monomers are predominant under the condition of incubation of ENTH domains with liposomes.

### Features of ENTH-induced membrane remodeling

Our simulation results suggest that the formation of membrane vesicles or tubules via ENTH‐domain-induced membrane remodeling of liposomes is a complex process that requires a balance between local ENTH domain concentration and sufficient time to grow the anisotropic ENTH domain lattice, for example, the formation of dimeric ENTH domain structure, and then larger ordered aggregates. For membrane tubulation, the first requirement is sufficient local concentration of stably bound ENTH domains (i.e., with H_0_ inserted). However, stable binding of an ENTH domain in the membrane will likely dramatically slow the ENTH domain diffusion rate; thus, a membrane-bound ENTH domain requires more time to encounter another ENTH domain. A sufficient amount of ENTH domains initially bound to the membrane will facilitate the process of dimer formation. Once ENTH domain dimers are formed, as observed from the EPR experiments, ENTH domain dimers may direct the formation of larger ordered ENTH domain aggregates, such as 4-mers, 8-mers, or larger oligomers. Continuing growth and lateral packing of such ordered aggregates can promote anisotropic spontaneous curvature. By contrast, if the local ENTH domain concentration is initially very high, stable PIP_2_ binding and insertion of H_0_ can drive membrane remodeling in an isotropic way to generate vesiculated structures before ENTH domains can arrange into an anisotropically ordered lattice.

It can be argued that the driving forces of the formation of ordered ENTH packing found in EPR measurements might be partially due to the experimental conditions by using preformed membrane tubules. This is possible, as it has been previously reported that preformed tubules can help crystallize proteins in a helical manner.^69^ Also echoing our hypothesized mechanism of ENTH tubulation described above, we suggest that inducing locally anisotropic curvature by locally anisotropic packing of ENTH domains is likely a precursor to initiate a positive feedback loop of ENTH domain anisotropic packing and to ultimately induce a large-scaled membrane tubulation process. A preformed membrane tubule condition likely reduces the energetic cost of ENTH domains creating an anisotropic curved environment and hence facilitates the positive feedback loop. Nevertheless, it is reasonable to conclude that an anisotropic packing of ENTH domains may represent an important feature of ENTH‐domain-induced membrane remodeling. Moreover, the preformed membrane tubule is a useful experimental design that allows us to investigate the oligomerized structure of membrane‐tubule-bound proteins such as ENTH. This is especially useful when the protein-bound membrane tubule structure is intrinsically unstable, as is the case for ENTH tubules.

## Summary and Conclusions

In this article, we have presented a study combining EPR spectroscopy with atomistic and CG MD simulations to investigate the binding and self-association of ENTH domains on membrane surfaces. The EPR experiments indicate that the ENTH domain H_0_ helix penetrates into the outer leaflet of the bilayer and forms an α‐helix in both membrane vesicles and preformed membrane tubes. From both EPR measurements and atomistic MD data, immersion depth measurements also indicate that H_0_ is located at the level of the lipid phosphate groups. The amphipathic nature of the H_0_ helix results in a structural motif through which to generate locally anisotropic membrane curvature via a wedge mechanism in that it does not penetrate too deeply into the bilayer hydrocarbon core, yet does not dislodge. In addition, atomistic MD simulations offer more detail on the ENTH domain–membrane binding interface. The results show that specific coordination between positively charged residues in the binding pocket and the PIP_2_ headgroup is likely important for the stabilization of the ENTH domain–PIP_2_ complex. This provides atomistic level insight into the origin of ENTH domain–PIP_2_ specificity.

In order to access larger length scales and longer time scales by simulations and to make a closer comparison with experiments, we developed and applied a systematic CG model in CG MD simulation to investigate how the ENTH domain interacts and self-associates on membrane vesicles and tubes. The results suggest that the arrangement of ENTH domains on the membrane is tightly coupled to local membrane morphologies, for example, membrane structure exhibiting anisotropic or isotropic membrane curvature. The origin of large-scale ENTH domain-dependent membrane tubulation resides in the collective organization of multiple ENTH domain dimers at sufficient density so that the collective effect of multiple, nearly parallel H_0_ helices results in a strong anisotropic spontaneous curvature inducing tubulation. Both EPR experiments and the CG simulations support the existence of ENTH domain dimers in the case of membrane tubes. Conversely, vesiculated structures appear to contain mainly ENTH domain monomers under the conditions studied, which is supported by both EPR experiment and CG MD simulations.

Utilizing the input from EPR experiments, molecular‐level details from the CG MD simulations lead us to suggest how self-association of ENTH domain may affect membrane remodeling pathways. We argue that the outcome of ENTH‐domain-induced membrane remodeling can be very sensitive to the initial concentration of ENTH domain on the membrane surface. An initially high density of ENTH domain may directly promote membrane vesiculation, whereas an initial, relatively lower density of ENTH domain binding may allow the growth of stable ENTH domain lattices and favors membrane tubulation. CG MD simulations support the possibility that the formation of stable ENTH dimers may play a role in facilitating membrane tubulation. The ENTH domain dimers may function to trigger the formation of a locally ordered ENTH domain aggregate, which in turn recruits more ENTH domains, resulting in an ever larger patch of anisotropically packed ENTH domain lattice. These results highlight the fact that combining EPR experiments and atomistic and MD simulations is a powerful approach to gain a deeper understanding of challenging membrane protein systems, which are often difficult to study via experimental or computational techniques independently.

## Materials and Methods

### Atomistic MD simulation setup

Atomistic MD simulations utilized a single ENTH domain bound to the bilayer solvated with explicit TIP3P water^70^ and sufficient Na^+^ counterions to maintain overall charge neutrality (Fig. 1a). The initial coordinates for the ENTH domain were taken from the crystal structure (Protein Data Bank code 1H0A).^16^ For the lipid bilayer, an all-atom 80/20 palmitoyl-oleoyl phosphatidylcholine/palmitoyl-oleoyl phosphatidylserine mixed bilayer is used. The mixed bilayer was generated as described in a previous work.^71^ The ENTH domain/lipid bilayer system was built in the following way: The binding of PIP_2_ headgroup and the formation of H_0_ is assumed for the ENTH–bilayer bound complex. To embed H_0_ to the lipid bilayer interface, we removed five lipids (four PC lipids and one PS lipid). The orientation of H_0_ is approximately parallel to the membrane surface as observed from EPR experiments. The initial coordinates for the PIP_2_ headgroup bound to the ENTH domain are taken from Ford *et al.*^16^

Subsequent solvation, minimization, and MD equilibration protocols were carried out as follows. After the ENTH domain was placed on the lipid bilayer surface, the system is solvated with TIP3P water and neutralized by Na^+^ counterions. The resulting simulated system possessed ~ 97,000 atoms. Initially, all C^α^ and three phosphorus atoms of the PIP_2_ headgroup were harmonically restrained with a 5 kcal/(mol Å^2^) force constant and a conjugate gradient minimization of 5000 steps was applied followed by heating to 310 K followed by 100 ps constant NPT equilibration. The following three stages and a total of 70 ns constant NPT equilibration were then performed to fully relax the interaction of the ENTH domain and bilayer. In the first stage of 10 ns constant NPT equilibration, restraints were only applied on all the C^α^ and phosphorus atoms of the PIP_2_ headgroup to relax the bilayer. For the second stage of 10 ns constant NPT equilibration, the restraints on C^α^ were released except for the H_0_ part (residue 1 to residue 15). Finally, 50 ns of unrestrained simulation was performed as the final stage of equilibration. A production run of 50 ns was then performed for data analysis and the parameterization of the ENTH CG model.

Atomistic MD simulations employed CHARMM22^72^ and CHARMM27^73^ force field parameters with the CMAP correction to describe the protein and the lipid–protein interactions, respectively. The parameterization for PIP_2_ lipid was as described in Lupyan *et al.*^74^ Simulations were performed under isothermal, isobaric conditions (constant NPT) and periodic boundary conditions. A Langevin thermostat with a damping coefficient of 0.5 ps^−^ ^1^ was used to maintain the system temperature at 310 K. The system pressure was maintained at 1 atm using a Langevin piston barostat.^75^ Semi-isotropic pressure coupling was employed to retain the square shape of lipid bilayer throughout the simulation. Short-range nonbonded interactions were truncated smoothly between 10 and 12 Å, and the particle mesh Ewald algorithm^76^ was used to compute long-range electrostatic interactions at every time step. All covalent bonds involving hydrogen were constrained by the SHAKE algorithm (or SETTLE for water),^77^ permitting an integration time step of 2 fs. System minimization, equilibration, and dynamics were performed using the NAMD 2.7b1 software package.^78^ System construction and image generation were performed by using the VMD 1.8.7 software package.^79^

### CG model for CG MD simulations

#### CG membrane model

The hybrid analytical systematic (HAS) CG lipid model utilized in this work has been previously described.^61^ Each lipid is modeled by a single-site Gay–Berne analytical model with an aspect ratio of 3:1 to mimic the lipid shape, with the symmetry along the long axis broken to capture the amphiphilic nature of the lipids. The in-plane component of lipid–lipid interaction was parameterized directly from atomistic MD data via the multiscale coarse-graining methodology.^80–82^ The HAS CG membrane retains several important features of the membrane, for example, reasonable bending modulus, area expansion modulus, and liquid‐like lipid diffusion coefficient.^61^ The mixed phosphatidylcholine/phosphatidylserine HAS membrane used in the current study has been previously developed; a more detailed description can be found elsewhere.^83^

#### CG ENTH model

A CG model of the ENTH domain, which retains the overall protein shape (Fig. 4b) and key membrane–protein interactions, was developed. The optimal locations of the CG sites were determined by the essential dynamics coarse-graining method^84^ based on the 50-ns atomistic MD trajectory of the ENTH bound to a lipid bilayer. The essential dynamics coarse‐graining method ensures that the locations of CG sites are variationally selected to optimally represent the low‐frequency dynamics as observed in atomistic MD simulations. The interactions between the CG sites within a single ENTH domain are represented by harmonic springs, parameterized by the heteroENM method.^85^ The strength of the harmonic springs obtained from the heteroENM method matches the thermal fluctuations of CG distances as mapped from atomistic MD data. Lennard-Jones (LJ) interactions were used to model all lipid–protein and protein–protein interactions as described in a previous work.^46^ Additional lipid–protein interactions were applied to membrane‐interacting sites to better reflect the difference from the non-membrane‐interacting sites on the protein. CG sites 1, 2, and 10 in Fig. 4b are categorized as membrane interaction sites, where CG sites 1 and 2 represent H_0_ and CG site 10 represents the arginine 114 loop (R114 loop). The H_0_–membrane interaction has a well depth of 4 kcal/mol. This value is chosen by employing an empirical transfer free energy calculation of partitioning an ENTH H_0_ (residues 1–15, sequence: MSTSSLRRQMKNIVH) from water to lipid bilayer interface using the software MPEx.^86^ In this calculation, N-terminal and C-terminal groups were selected as “NH3+” and “CONH2” groups, respectively. By varying total helicity of ENTH H_0_ from 85% to 100%, the free energy ranges from − 3.4 to − 4.3 kcal/mol; this indicates that partitioning the ENTH H_0_ sequence into bilayer interface as an α-helix form is a thermodynamically favorable process. It should be noted that previously reported metadynamics simulations to fold and insert the endophilin H_0_ helices into a membrane^20^ favors the folded state by 4.8 kcal/mol with respect to the unfolded state, and this value is comparable to what we have used here. For the interaction between the R114 loop and membrane, a well depth of 1.2 kcal/mol was applied. This interaction is obtained from atomistic MD trajectories by constructing the potential of mean force (PMF) as a function of distance between the center of mass of the residues within the CG site of the R114 loop (residues 112 to 116) and the phosphorus atom of the lipid headgroups. The PMF is computed by Boltzmann inversion^87^ of the radial distribution function, as previously described.^46^ Finally, 10% of the HAS lipid headgroups, the same fraction of PIP_2_ lipids used in the EPR experiments, were given an additional interaction with the PIP_2_ binding pocket of the ENTH domain to account for the specific interaction between the PIP_2_ headgroup and the ENTH domain binding pocket. The location of the PIP_2_ binding pocket was defined by the crystal structure in which the positively charged residues interact with the 4-phosphate group of the PIP_2_ headgroup. This interaction has a well depth of 2.4 kcal/mol. The strength of this interaction is guided by experimental data to account for high binding affinity of ENTH for the PIP_2_ headgroup.^16^

### CG MD simulations setup: ENTH-bound membrane tubules and membrane vesicles

The systems of ENTH-bound membrane tubes and vesicle were simulated to mimic the EPR experimental conditions, with the diameters of the tubules and vesicles of the simulation systems chosen to match experimental length scales. The 16‐nm tubule diameter was chosen for ENTH domain-bound lipid tube systems based on the diameter of ENTH domain-tubulated structures reported in Ford *et al.*^16^ The diameter of vesicles (50 nm) used in CG simulations was chosen based on the experimental observation that small vesicles are the dominant products during incubation of ENTH domains with liposomes.

#### ENTH-bound membrane type A and type B tubes

Type A tubes extend across periodic boundaries while type B tubes are confined to one CG MD cell with truncated tube ends (cf. Fig. 6b). In the type A tube, a 50-nm‐long tube with a 16‐nm diameter was constructed with an ENTH domain lattice on it. The type A tube is designed to study the stability of the proposed ENTH domain lattice on membrane tube structure with anisotropic local curvature and also to help identify important factors for ENTH domain lattice stability. It should be noted that the protein lattice on type A tube does not interact with neighboring images of itself, only lipids do. The type A tube system consists of a total of 6510 CG lipids and 3072 CG protein sites, and this effectively represents a total of over 12 million atoms, including water solvent. For the type B tube, a 120-nm‐long tube with a 16‐nm diameter was built. The same ENTH domain lattice was built on the type B membrane tube, but neither end of the tube interacts with its own periodic boundary image and is truncated on both ends. The longer type B tube is designed to investigate how the stability of the protein lattice can affect the stability of the overall membrane tube. The type B tube system consists of a total of 16,485 CG lipids and 8960 CG protein sites and is equivalent to the total of over 35 million atoms, including water solvent.

It should be noted that the initial interactions between ENTH domains are tuned so that ENTH domains do not condense into an agglomerated phase. In order to account for the observation of an ordered structure found in EPR experiments and the identification of hydrophobic contacts mentioned above (V50 and V51 hydrophobic patch), we included two additional LJ interactions. First, to reflect the EPR observation of the formation of dimeric structure and larger aggregates (4-mer), 2‐kcal/mol additional interactions were included between CG site 1 and CG site 2 for the interactions between ENTH domains. Regarding the consideration of interaction strength between dimers mediated by the hydrophobic patch (discussed in Results), a 2‐kcal/mol additional interaction was applied to CG site 5 (Fig. 4b). It has been previously reported that association of two isobutane (designed to mimic the valine residue) exhibits around 1 kcal/mol well depth via a PMF calculation from the work of Makowski *et al*.^88^ In the present ENTH domain system, there are two valine residues for each ENTH domain involved for this hydrophobic interaction.

#### ENTH-bound membrane vesicle

A 50-nm‐diameter vesicle was created with 20,998 HAS CG lipids^61^; the number of lipids in the inner and outer leaflets was chosen in order to balance the lipid densities in each leaflet. The single ENTH domain was then replicated and radially arranged, and the ENTH domain H_0_ CG sites in each were initially positioned 0.5 nm off the membrane surface. A total of 441 and 438 ENTH domains were used in the original and the control membrane vesicle CG MD simulations, respectively. There were around a total of 28,000 CG sites in the simulations, effectively representing over 35 million atoms, including solvent.

### CG MD simulation protocol

CG MD simulations were carried out with the TANTALUS code developed in the Voth group.^46^ After gradually increasing the system temperature and time step, the simulations were stable at 300 K and with a time step of 0.001 ps. (Note that there is no direct correspondence between CG time and atomistic time; hence, this time step likely represents several orders of magnitude longer in atomistic time scales.) All CG simulations were carried under the constant NVT ensemble. The system temperature was set to 300 K and coupled to a thermal bath by the Nose–Hoover method.^89,90^ The equations of motion of the CG simulations were integrated with the velocity Verlet algorithm.

### Generation of epsin 1 ENTH (1–164) cysteine mutants

Rat epsin 1 ENTH domain was cloned into plasmid pGEX4T2. The native cysteine at position 96 was mutated to serine by site-directed mutagenesis. Specific cysteine mutations were introduced individually into the cysteine-less background clone by mutagenesis and confirmed by DNA sequencing.

### Purification of wild type and mutant rat epsin 1 ENTH

The purification of rat epsin 1 ENTH domain has been described previously (see Supplementary Information of Ref. 16). Briefly, rat epsin 1 ENTH and its mutants were generated as N-terminal glutathione *S*-transferase fusion proteins. Proteins were expressed using *Escherichia coli* BL21 (DE3) cells and induced overnight. The bacterial lysate was incubated with glutathione–Sepharose beads, washed with buffer, and then treated with thrombin to release the protein of interest. Proteins were loaded onto an ion‐exchange column, eluted with 20 mM Hepes, pH 7.4, 75 mM NaCl, and 2 mM DTT. The protein of interest came out in the flow-through. The protein was further purified by gel filtration. The present study uses the same construct as the original crystallography study, including an additional N-terminal GSPGIH sequence due to thrombin cleavage.

### Preparation of liposomes

Liposome preparation has been described previously.^16^ Lipids were obtained from Avanti Polar Lipids (Birmingham, AL). We mixed 10% cholesterol/40% phosphatidylcholine/40% phosphatidylethanolamine/10% phosphatidylinositol-4,5-bisphosphate (wt/wt) in a test tube and dried it using nitrogen gas. The dried lipid was desiccated overnight, hydrated with 20 mM Hepes, pH 7.4, and 150 mM NaCl. Lipid samples were briefly bath sonicated and then extruded through 0.4‐μm polycarbonate filters (Whatman) using a mini-extruder (Avanti).

### Preparation of premade tubes

Tubes are composed of 10% cholesterol/40% phosphatidylcholine/40% galactocerebrosides/10% phosphatidylinositol-4,5-bisphosphate. Lipids were obtained from Avanti Polar Lipids, while the galactocerebrosides were purchased from Sigma. The procedure for tube preparation is the same as for liposome preparation (see above).

### Spin labeling of single‐cysteine proteins

DTT was added to protein samples to a final concentration of 1 mM. DTT was removed by size exclusion using PD-10 columns (GE Healthcare) in 20 mM Hepes, pH 7.4, and 150 mM NaCl buffer. Five times molar excess of the MTSL spin label (1-oxyl-2,2,5,5-tetramethylpyrroline-3-methyl) was incubated with the protein samples for 30 min at room temperature (RT). Excess spin label was removed by size exclusion using PD-10 columns.

### Sedimentation assays and EPR experiments

Ten micromolar rat epsin 1 ENTH domain is incubated with 60 μg liposomes or premade tubes in 100 μl total volume. After a 10-min incubation at RT, samples were centrifuged at 152,800***g*** for 20 min to separate liposome bound or tube bound from unbound. EPR spectra were measured using a Bruker EMX spectrometer fitted with a dielectric resonator. Spectra were measured at 4 mW incident microwave power. The O_2_ and NiEDDA П parameters were measured using the power saturation method previously described.^91,92^ The final concentration of NiEDDA was 10 mM, while the final concentration for O_2_ was the concentration in equilibrium at RT.

To test for spin–spin interaction of singly labeled epsin derivatives, we recorded EPR spectra at a scan width of 150 Gauss and we compared spectra for fully labeled proteins with those from spin‐diluted proteins in which mixtures of 33% labeled and 67% unlabeled protein were used using the same protein‐to‐lipid ratios and concentrations as described above. All spectra were then normalized to the same number of spins by double integration. Distance estimates were obtained using the deconvolution methods^55,56^ as implemented by Altenbach *et al.*^56^ using an automated fitting routine and a Gaussian distribution (Shortdistances V21_041) developed by Dr. Christian Altenbach using LabView (National Instruments) and available for download.

### Depth calibration

To calibrate the immersion depth of R1 at different sites, we used the previously established relationship *d*[Å] = a*Φ + *b*, where Φ = LN(ΠO_2_/ΠNiEDDA).^91^ To obtain the parameters for *a* and *b*, we used liposomes containing 10% phosphatidylinositol-4,5-bisphosphate as well as 1-palmitoyl-2-stearoyl(*n*-DOXYL)-*sn*-glycero-3-phosphocholine (Avanti Polar Lipids) with the spin label attached at the 5, 7, 10, and 12 positions on the acyl chains.^91^ For the spin-labeled phosphatidylcholines in liposomes containing epsin 1 ENTH domain under the conditions described above, we found that *a* = 3.5 and *b* = 0.37.
