# Supplementary material for: Membrane Binding and Self-Association of the Epsin N-Terminal Homology Domain
Source: J Mol Biol. 2012 Nov 9;423(5):800–17. doi: 10.1016/j.jmb.2012.08.010 (PMC3682188; doi:10.1016/j.jmb.2012.08.010)
Supplement: Supplementary file 1 — Supplementary materials [file mmc1.pdf]

Supplementary Data for

Membrane Binding and Self-Association of the Epsin N-Terminal Homology Domain

Chun-Liang Lai[§1], Christine C. Jao[§2], Edward Lyman[3], Jennifer L. Gallop[4], Brian J. Peter[4], Harvey T. McMahon[4], Ralf Langen[2*], and Gregory A. Voth[1*]

[1]*Department of Chemistry, Institute of Biophysical Dynamics, James Franck Institute, and Computation Institute, University of Chicago, 5735 S Ellis Ave., Chicago, IL 60637, USA.*
[2]*Zilkha Neurogenetic Institute, University of Southern California, 1501 San Pablo Street, Los Angeles, CA 90033*
[3] *Department of Physics and Astrophysics, and Department of Chemistry and Biochemistry, University of Delaware, Newark, DE 19716*
[4]*MRC Laboratory of Molecular Biology, University of Cambridge, Hills Road, Cambridge, CB2 2QH, UK.*

The N-terminus of the epsin 1 ENTH domain becomes structured upon membrane interaction

In order to test whether the N-terminus of epsin 1 undergoes a conformational change upon membrane interaction, we generated spin labeled derivatives of the epsin 1 ENTH domain and recorded their EPR spectra in solution and when bound to liposomes containing 10% phosphatidylinositol 4,5-bisphosphate (PIP_2). As illustrated in **Fig. S1**, all EPR spectra for the singly labeled N-terminal sites (positions 4-14) give rise to sharp and narrowly spaced lines of large amplitude when no liposomes are present (**Fig. S1**, black dashed line). These spectra indicate very high mobility and are in agreement with the highly dynamic structure of the N-terminus shown by NMR and X-ray crystallography.[1,2]

Next, the spin labeled derivatives were bound to liposomes containing 10% PIP_2. All samples bound to membranes as assayed by co-sedimentation studies[2] and retained their ability to induce membrane curvature. Under the present conditions, the epsin derivatives caused tubulation as well as vesiculation with the latter being the predominant species as judged by negative stain electron microscopy (EM data not shown). It should be noted that the 7R1 derivative was not generated because this residue is involved in coordinating headgroup binding via two contacts.[2] In the presence of liposomes containing 10% PIP_2 (**Fig. S1**, red trace), all spectra exhibit pronounced line broadening and concomitant reduction in signal amplitude in agreement with a previous study.[3] To visualize the line shape changes of the R1 sidechain in the liposome-bound form, all spectra measured in the presence of membrane are shown at five-fold increased magnification. Most spectra display the characteristic line shapes observed for surface sites in a-helical structures. The spectrum for 8R1 is more complex as it has an additional sharp component (**Fig. S1**, green trace) that could readily be subtracted using the spectrum of the unbound protein and the resulting spectrum is indicated by the red trace. According to the crystal structure of epsin 1, the ENTH domain in the presence of the headgroup IP_3,[2] R8 forms a direct contact with the headgroup and this mutation is likely to reduce (but not abolish) the binding affinity. More strongly immobilized components are observed at a few sites (5R1, 8R1, 9R1,

and 12R1, **Fig. S1**, see arrow) suggesting that the R1 motion is restricted at these sites, possibly due to contacts with nearby residues or headgroups (see below). These spectral changes indicate structural ordering in residues 4 through 14 in the presence of liposomes.

Secondary structure and membrane topology of liposome-bound H_0

To further characterize the structure and topology of the membrane-bound form of the ENTH domain, we measured the accessibilities of the R1 side chain to the paramagnetic colliders, O_2 and NiEDDA. The nonpolar O_2 preferentially partitions in the hydrophobic environment of the membrane while the more polar NiEDDA preferentially partitions in the solvent. Thus, membrane-exposed residues are more O_2 accessible while solvent-exposed sites are more accessible to NiEDDA.[4-6] When plotted as a function of residue number, the accessibility parameters (ΠO_2 and ΠNiEDDA) exhibit periodic and out-of-phase oscillations that are highly characteristic of regions that are exposed to membrane on one side and to the aqueous environment on the other. The accessibility data can be conveniently summarized by the depth parameter Φ (where $\Phi = LN [\Pi O_2/\Pi NiEDDA]$) which increases proportionally with increasing immersion depth in the bilayer. As shown in **Fig. 1e**, Φ oscillates periodically with respect to residue number and the period of this oscillation corresponds to that of an a-helix. In this helix, residues 6, 10, 13 and 14 are lipid-exposed (magenta filled circles) while residues 5, 8, and 12 are the most solvent accessible (green filled circles). Moreover, when plotted using a helical wheel representation (**Fig. 1f**), the lipid-exposed residues (magenta) cluster together and lie on the opposite side of the solvent accessible residues (green).

Previous studies using SDSL demonstrated that Φ values are proportional to the depth of membrane insertion, and that this depth can be calibrated using spin-labeled derivatives of phospholipids.[6] Based on the calibration described in Materials and Methods, the average immersion depth of the lipid-facing R1 side chains (Φ maxima) is on the order of 8 Å. Inasmuch as the nitroxide moiety of the R1 sidechain is typically within 7-10 Å of the center of the helix,[7] we can estimate that the center of the epsin 1 ENTH domain N-terminal helix is, therefore, at the level of the headgroups where it is likely to exert membrane "wedge" effects, leading to membrane curvature generation.

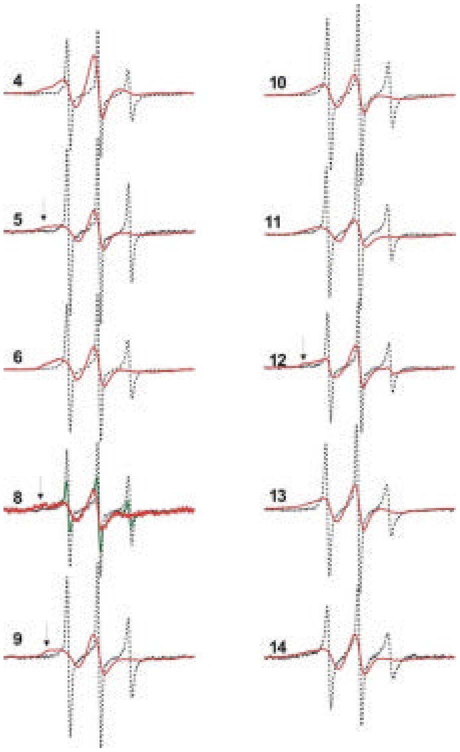

Fig. S1. X-band EPR spectra of R1-labeled epsin 1 ENTH domain derivatives in buffer in absence (black dashed line) and presence of lipid (red solid line). The green trace for 8R1 is the experimentally determined spectrum prior to subtraction of the spectrum for the soluble form. All spectra were normalized to the same number of spins and the spectra for the membrane-bound forms are shown at five-fold magnification. The scan width for all spectra is 100 Gauss.

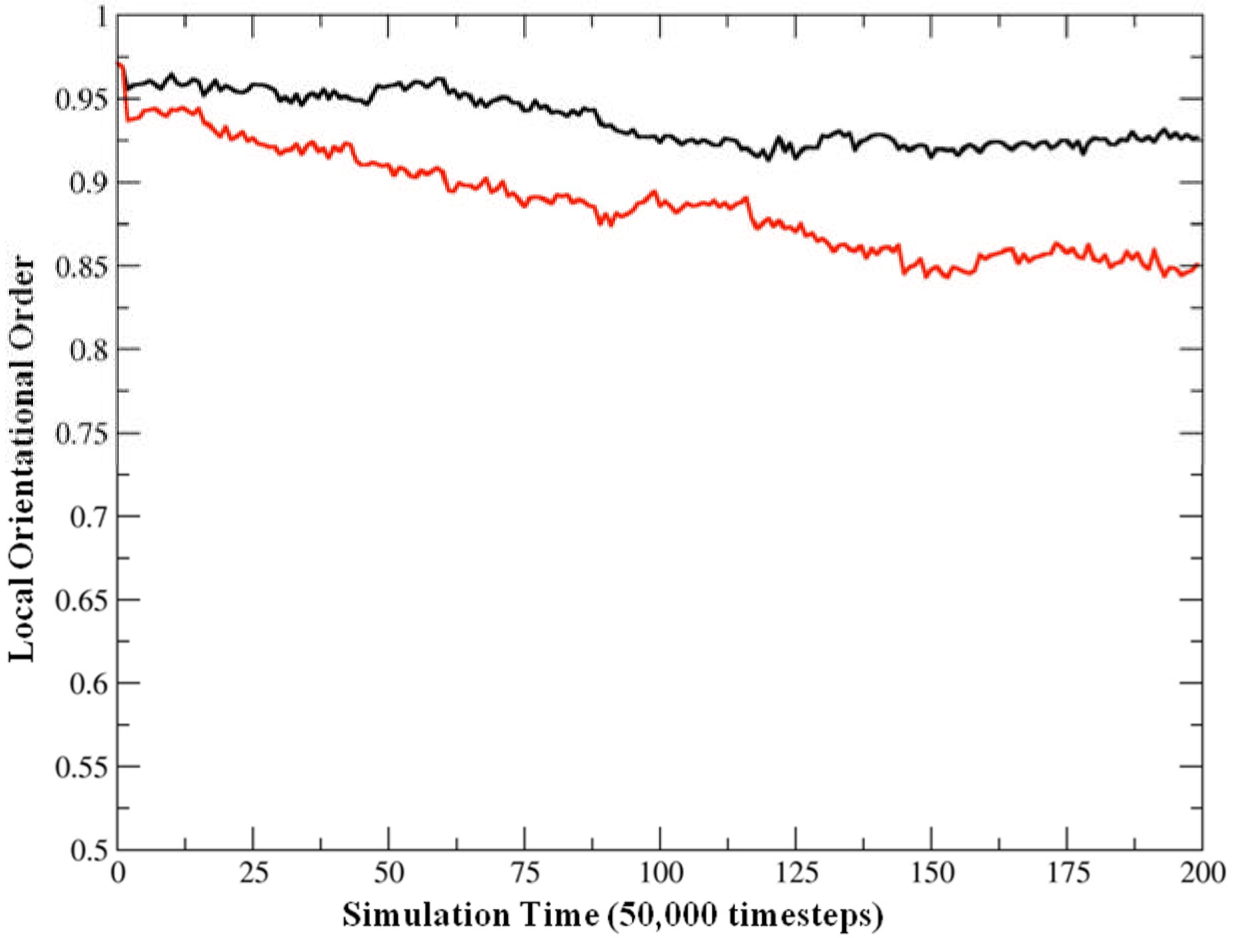

Fig. S2. Time dependence of local orientational order parameter for the type A tube systems. Local orientational order parameter is defined to quantify relative orientation ENTH with respect to neighboring ENTHs. This order parameter is calculated as the dot product of orientations between two H_0 of ENTH, averaged over all pairs that are within 5 nm, and indicates the average alignment among local neighborhoods of ENTHs. A perfectly aligned neighborhood has an averaged value of 1. The control experiment (red line) is used to study the condition in the absence of additional attractive interactions between CG site 1 and CG sites 2 for inter-protein interactions. Details of the CG simulation system setup can be found in the Materials and Methods section of the main text.

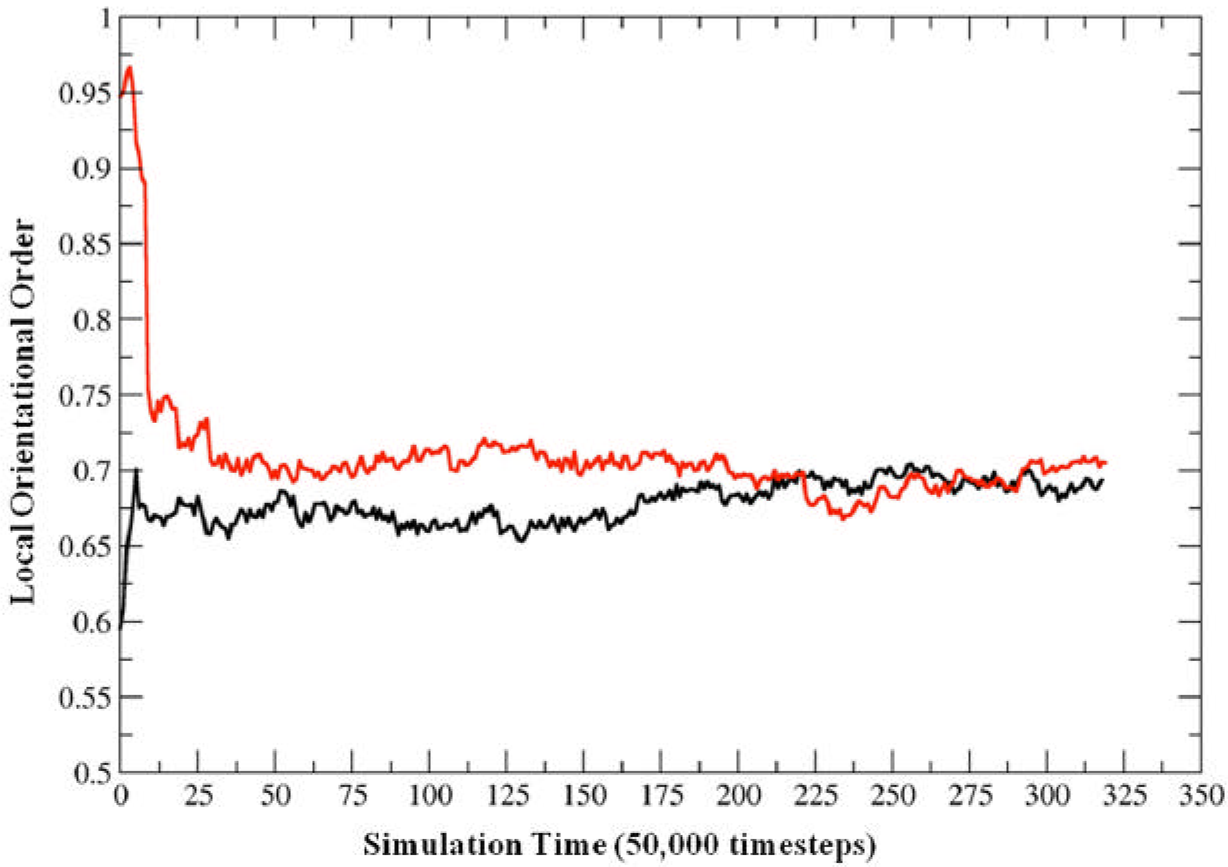

Fig. S3. Time dependence of local orientational order parameter for the membrane vesicle system. Local orientational order parameter is defined to quantify relative orientation ENTH with respect to neighboring ENTHs. This order parameter is calculated as the dot product of orientations between two H_0 of ENTH, averaged over all pairs that are within 5 nm, and indicates the average alignment among local neighborhoods of ENTHs. A perfectly aligned neighborhood has an averaged value of 1. The black line represents the system with randomly arranging ENTH monomers on the surface of membrane vesicle, whereas the red line is the control experiment starting off by randomly arranging ENTH dimers on the surface of the membrane vesicle. Details of the CG simulation system setup can be found in the Materials and Methods section of the main text.

Table S1. Type B tube is divided into either five or seven segments and computed averaged local orientational order parameter for each individual segment. Data used to compute averaged local orientational order parameter were taken from the last 4.5M timesteps of trajectory of the type B tube system. The errors of the local orientational order parameter results in this table fall within a range +/- 0.01. The error was estimated by calculating the standard deviation of the averaged order parameter over 1.5M trajectory blocks over the last 4.5M timesteps of simulation trajectory.

	**Five segments**	**Seven segments**
**1**	0.87	0.86
**2**	0.91	0.87
**3**	0.90	0.93
**4**	0.92	0.89
**5**	0.84	0.94
**6**		0.90
**7**		0.90

References:

1. Itoh, T., Koshiba, S., Kigawa, T., Kikuchi, A., Yokoyama, S. & Takenawa, T. (2001). Role of the ENTH domain in phosphatidylinositol-4,5-bisphosphate binding and endocytosis. *Science,* **291**, 1047-1051.
2. Ford, M. G. J., Mills, I. G., Peter, B. J., Vallis, Y., Praefcke, G. J. K., Evans, P. R. & McMahon, H. T. (2002). Curvature of clathrin-coated pits driven by epsin. *Nature,* **419**, 361-366.
3. Kweon, D. H., Shin, Y. K., Shin, J. Y., Lee, J. H., Lee, J. B., Seo, J. H. & Kim, Y. S. (2006). Membrane topology of helix 0 of the Epsin N-terminal homology domain. *Mol. Cells,* **21**, 428-435.
4. Hubbell, W. L., Gross, A., Langen, R. & Lietzow, M. A. (1998). Recent advances in site-directed spin labeling of proteins. *Curr. Opin. Struct. Biol.* **8**, 649-56.
5. Hubbell, W. L., Cafiso, D. S. & Altenbach, C. (2000). Identifying conformational changes with site-directed spin labeling. *Nat. Struct. Biol.* **7**, 735-739.
6. Altenbach, C., Greenhalgh, D. A., Khorana, H. G. & Hubbell, W. L. (1994). A collision gradient method to determine the immersion depth of nitroxides in lipid bilayers: application to spin-labeled mutants of bacteriorhodopsin. *Proc. Natl Acad. Sci. USA,* **91**, 1667-1671.
7. Langen, R., Oh, K. J., Cascio, D. & Hubbell, W. L. (2000). Crystal structures of spin labeled T4 lysozyme mutants: implications for the interpretation of EPR spectra in terms of structure. *Biochemistry,* **39**, 8396-8405.
